# Freedom to Trade and Safeguarding the National Health Service (NHS) - Did the UK-Australia Free Trade Agreement Safeguard the NHS?

**DOI:** 10.1177/27551938251411268

**Published:** 2026-02-10

**Authors:** Alison McCallum, Meri Koivusalo, Silke Trommer

**Affiliations:** 1 Centre for Population Health Sciences, Usher Institute, University of Edinburgh, Edinburgh, UK; 2 528748 Faculty of Social Science, Tampere University, Tampere, Finland; 3 Comparative Public Policy 6061 , Politics, University of Manchester, Manchester, UK

**Keywords:** health systems, UK, Australia, NHS, trade, health policy

## Abstract

The United Kingdom (UK) government has claimed that the National Health Service (NHS) was safeguarded throughout the negotiations of the Free Trade Agreement (FTA) with Australia. This article examines if this was achieved. We used public documents on the UK-Australia FTA, including impact assessments, public consultations, and the text of the negotiated agreement. Analytically, we drew on process tracing to assess whether and how the NHS was (i) included in impact statements and assessments, (ii) excluded from the agreement, and (iii) addressed in key chapters, focusing on (a) to what extent NHS safeguarding differs from that of other comparable public policy prerogatives, such as audiovisual services covering national broadcasting, (b) health-related limitations set by Australia, and (c) using the legal obligations of World Trade Organisation (WTO) membership as the null option. Our analysis shows that the NHS was not fully excluded from the trade agreement on the ground of the negotiated agreement compared with the null option of WTO provisions, including for services and public health protection. This would have been possible, but in several chapters was not considered. There was no overarching exclusion and the NHS gained less comprehensive exclusion in comparison to audiovisual services and, in some chapters, to the health-related restrictions set by Australia.

## Background

While global trade politics are in turmoil, this should not distract our focus on what free trade deals contain and how more aggressive trade and commercial policies could be used to enhance investor or corporate priorities as set in negotiated trade agreements between countries. Trade agreements – or deals – cover not just tariffs, but wider questions on services, public procurement, and protection of intellectual property rights and investors.^1^ These remain of interest to major global industries, including with respect to pharmaceuticals, health and health-related services, and trade and investment in fields of importance to national health systems governance. Contemporary trade agreements extend to beyond-the-border impacts on services, protection of intellectual property rights (IPR) and investment, and to government procurement and have wider domestic impacts, including upon the public sector, non-trade policy priorities, and policy space for regulation at national level.^2^ We define policy space as the freedom, scope, and mechanisms available to governments from which they choose, design, and implement public policies to fulfil their aims.^2^ A key question for trade agreements is to what extent they may – or may not – provide contractual power for the benefit of major industries against wider public interest and policy priorities.

While trade agreements can impact on the wider determinants of health and essential services, in this article we focus more specifically on the claim of keeping the NHS across the United Kingdom (UK) outside of the scope of Free Trade Agreements (FTAs). We examine if “taking back control” resulted in full consideration of the NHS as part of trade negotiations in the context of negotiations on UK-Australia FTA, the first FTA negotiated from scratch following Brexit. Previous research has exposed potential risks to the NHS and public health related to Brexit, Brexit-related trade agreements, and a potential US-UK FTA.^3–5^ The UK-Australia FTA provides insights on the intersection between trade and health and sets a precedent for future negotiations, including implications for the NHS. It thus provides an excellent case study for analysis due to government claims that the NHS has been safeguarded.

When submitting the agreement to the House of Commons, the then UK Secretary of State for International Trade declared: “*We have […] secured protections relevant to the NHS and Australia's health system in the agreement, which keep the NHS out of scope of the agreement*”.^6^ Our study assesses to what extent this claim of keeping the NHS out of scope of the agreement has been realised in the negotiated text. The then Minister for Trade later reiterated:

“*this and our other free trade agreements do not, and will not, cover healthcare services in the UK—neither will they threaten the standard of care nor the Government's ability to decide how we and this* *Parliament organise our healthcare services in this country in the best way for patients*”.^7^

We further assess to what extent the claim of keeping the NHS out of scope of the agreement has been realised in the negotiated text.

Our wider theoretical framework is in line with Rodrik's^1^ emphasis on “what trade agreements do” and focuses on trade agreements as legal agreements and means of governance. In the light of research on trade and health we know that chapters on services, investment, competition, and government procurement may affect health systems and regulatory policy space.^2,8–13^ IPRs and trade secrets impact access to vaccines, pharmaceuticals, and technologies.^14–17^ Rules on state-owned enterprises (SOE) may also be relevant to the NHS.^18^ We can also anticipate increasing relevance of trade in digital services to how health systems operate and how health services are provided. We further examine whether the UK's commitments in the FTA go beyond World Trade Organisation (WTO) requirements, which is expected to form the base-level of negotiations.

We recognize that each of the UK countries has its own primary NHS legislation. Organisation and governance of the four health systems also varies. Responsibility for trade and investment agreements, however, is held at UK level. We therefore highlighted areas likely to be relevant to consideration of the extent to which trade and investment agreements might impact on the safeguarding of the NHS. Health care reforms undertaken since the 1990s in the UK have provided scope for outsourcing and private sector engagement in the NHS,^19–21^ but reforms have also restricted this as in Scotland's NHS Reform (Scotland) Act in 2004.^22^ The NHS in England has continued the purchaser provider split with most specialist services provided by NHS Trusts and extended market-based reforms with the Health and Social Care Act 2012.^23–25^ Foreign providers already hold contracts under the NHS.^26–27^ The provision of specialist public health functions remains an NHS and non-departmental body function in Scotland, Northern Ireland and Wales, however, the Health and Social Care Act 2012 also transferred provision and commissioning of public health functions in England from the NHS to Local Authorities. The extent to which safeguarding the NHS encompasses services commissioned or delivered through public health departments in England is not clear.^28–29^

## Material and Methods

Our sources for this case were publicly available documents on UK-Australia trade negotiations, impact assessments, consultations, and the UK-Australia FTA text.^30–31^ The FTA texts were downloaded from UK and Australian government websites. Using documentary analysis preparing, extracting, analysing, and summarising findings, examining the relationships between the various documents,^32^ and process–tracing^33–34^ we analysed to what extent the NHS is safeguarded and “out of scope” of the agreement on the ground of the negotiation results, ie the final text. The analytical framework was based on first examining if and how the NHS was considered as part of impact assessment of the agreement and consultations related to the negotiation. This was to give insight on the extent to which safeguarding NHS was part of wider priorities. We then moved to process tracing to test first if NHS safeguarding had been secured through more general exclusion from the agreement. If not, we then proceeded to examine if this had been done through chapter-based exclusions in each relevant chapter. A third avenue was to examine if consideration of the NHS was equal or more stringent than a comparable other area for safeguarding public policy prerogatives, for example audiovisual services covering national broadcasting services, such as the British Broadcasting service Corporation (BBC). Audiovisual services provide a good reference as they represent services that European Union member states have actively sought to exclude from trade agreements governing services trade.^35^ We then compared whether safeguards were more stringent than in the WTO agreements, which we used as the baseline for bilateral agreements and null option for our comparison, as WTO rules apply in the absence of an FTA. Finally, as trade agreements are negotiated between two governments, we examined if the UK safeguarded the NHS more comprehensively than Australia. The basic hypothesis for this assessment was that, as the UK is a larger economy than Australia and a high priority to the UK, the negotiations would have given it the scope to safeguard NHS as far as it desired.

We thus examined (i) UK impact assessments of the agreement (scrutiny of potential impacts); and (ii) overarching NHS clauses or exceptions to indicate if the NHS was safeguarded as part of wider measures; or (iii) chapter-by-chapter NHS clauses or exceptions. We then focused on how NHS exclusions relate to (iv) other public policy concerns, such as audiovisual services; (v) Australian health system exclusions; and (vi) WTO provisions.

## Results

The UK takes NHS-relevant commitments across several chapters, annexes and side-letters to the agreement. We discuss the impact assessment (IA), general clauses, results for each chapter, and cumulative effects below.

### Impact Assessment

The UK government's impact assessment states: “*Throughout the FTA the UK has secured exceptions and exclusions for the NHS – our National Health Service and the services it provides will never be on the table*”,^36^ ^p 15^. Devolved governments largely took these statements at face value, although the Welsh submission identified 10 chapters with direct implications for health.^37^

In contrast to the strong statement on protection of the NHS in the impact assessment, the UK government impact assessment does not examine NHS exclusion mechanisms in the agreement. Only the statement and overview discuss the NHS. The impact assessment considers public health concerns, including air pollution,^38^ ^p.^^39,42^ ^p.^^48^, therefore not omitting health considerations entirely. The NHS was thus not omitted due to no consideration of health or health system implications. This was also highlighted in the consultations, where “*a significant number of stakeholders”* in public consultations articulated the importance of protecting public services and safeguarding the NHS,^40^ ^p.^^17^ Lack of knowledge of public concern does not, therefore, explain why potential risks to the NHS were not evaluated as part of the impact assessment. This conflicts with the statement and overview, which are not based on the impact assessment process. The impact assessment could have been extended to include direct and indirect implications for the NHS, including analysis on the ground of previously published work on assessing implications,^3–5^ but this was not done.

A general clause in the FTA that excluded the NHS and public health services could explain omission from the scope of the impact assessment. However, we could not find a general clause excluding the NHS fully from the FTA.

### General Clauses

The FTA's preamble recognises each party's right to protect and promote public health. However, preambles include secondary clauses, indicating the signatories' intentions. It is the substantive chapters of the FTA that shape *how* each party can protect and promote public health. Art 31.7 under the agreement's General Exceptions is entitled “The National Health Service and Australia's Health System”. However, it does not exclude the NHS from the agreement but affirms NHS-related exceptions in “Chapter 8 (Cross-Border Trade in Services), Chapter 13 (Investment), Chapter 15 (Intellectual Property), Chapter 16 (Government Procurement), Annex I (Schedules of Non-Conforming Measures for Services and Investment) and Annex II (Schedules of Non-Conforming Measures for Services and Investment)”. In so far as Article 31.7 reiterates the NHS-related exemptions in specific chapters of the agreement, the provision makes clear that assessing to what extent the NHS lays outside of the scope of the agreement requires a chapter-by-chapter analysis. Such an analysis reveals the scope of the NHS-specific exemptions, as well as NHS-relevant activities that are subject to the agreement's provisions.

A general letter added to the agreement sets out further public health cooperation.^41^ While Art 22.3.6 resists weakening environmental protections to encourage trade and investment, there is no similar direction regarding human health and universal healthcare free at the point of use.

A general exclusion clause could have been made to exclude any services and goods directly relevant to sustainable financing and organisation of the NHS, more narrowly to exclude the NHS, or more explicitly to exclude the NHS, including any measures concerning contracted and subcontracted services, procurement of goods and health-related products, and incentives for R&D for health-related products. Furthermore, exclusion clauses could have encompassed the entire FTA or been included in *relevant* chapters, as for other public policy prerogatives such as audiovisual services.

### Chapter by Chapter Analysis

To determine the extent of NHS safeguarding, therefore, it is necessary to examine the agreement chapter-by-chapter. Table 1 juxtaposes UK-Australia FTA chapters where the NHS falls directly or indirectly within their remit with emphasis on health service provision. Other chapters may also impact health, particularly the social, environmental, and commercial determinants of health (Table 1).

**Table 1.** The NHS in the UK-Australia FTA.

NHS Directly in Scope	NHS Indirectly Affected
Chapters 8, 10 & 11: Services Chapter 13: Investment Chapter 14: Digital Trade Chapter 16: Government Procurement Chapter 18: State-Owned Enterprises Chapter 26: Good Regulatory Practice Chapter 15: Intellectual Property Rights Chapter 17: Competition policy and consumer protection	Chapter 9: Financial Services Chapter 6: Sanitary and Phytosanitary Measures Chapter 7: Technical Barriers to Trade Chapter 20: Innovation Chapter 25: Animal Welfare and Antimicrobial resistance

### Services

The agreement includes central, regional, and local levels of government and non-governmental bodies (Art 8.1). Therefore, any health and social care services provided via devolved, or council-level public authorities are in scope. Unless they are explicitly excluded, the ‘negative list approach’, means that all services and service sectors are included.

Exclusions feature within chapters and the party's schedules in Annexes. Financial services, government procurement, services supplied in the exercise of government authorities, government subsidies or grants, audio-visual services, and most air services are excluded from cross-border service liberalisation (Art 8.2) as well as recognition of some professional qualifications (Art 10.2). However, the narrow focus of ‘services supplied in the exercise of government authority’ (Art 8.1 and Art 8.2) means this definition is unlikely to protect the NHS.^42–43^ While professional health services are not explicitly listed under recognition of professional qualifications, Art 10.2 includes “other types of professional services”, under which health services could fall.

The ‘ratchet clause’ (Art 8.7.1(c)) automatically liberalises services if legislation changes. Annexes I and II exclude specified sectors and activities from this mechanism and cover existing and future legislation. There are no directly relevant NHS reservations for services in the UK's Annex I. The Australian Annex I specifies ownership requirements for pharmacies and for the biotech company CSL, a previously government-owned body.

Annex II includes some measures that may limit application of the ‘ratchet clause’. The Explanatory Note to Annex II specifies in paragraph 5 that measures relating to licensing, training and qualification can continue to apply, including requirements for specific professions, or to satisfy universal service obligations. Paragraph 6 also carves out measures relating to competition law and those “seeking to ensure the conservation and protection of natural resources and the environment”, but not health, public health or the NHS.

The UK Government could still have excluded the NHS extensively under Annex II, as this provides exceptions for future measures. In Annex II the UK reserves the right for any measures in relation to health services for cross-border trade, however privately funded hospital services are included under investment and domestic regulation obligations. In Annex II-9 the right to set conditions if services are sold is reserved. The UK also reserves the right to regulate all health-related professional services where the service crosses the border, and to regulate retail sales of medical and orthopaedic goods and pharmaceutical goods and services. Australia does not have similar reservations for pharmacists and retail of pharmaceutical goods but has more explicit reservations regarding data on health and children, blood and blood products, and the pharmaceutical benefits scheme.

### Investment

Given the narrow definition of “activities carried out in the exercise of governmental authority” (Art 13.1), chapter 13 covers investments in the health and care sectors, except health insurance (Art 13.3), and intellectual property rights (IPR). The chapter binds government agencies at all levels, from central to local (Art 13.2). The agreement provides free market access (Art 13.4), national treatment (Art 13.5) and third-party rights (Art 13.6) for investors. Governments can generally not limit investors, investments, or employee numbers, impose a particular legal entity, or set performance requirements on investors (Art 13.11). However, the agreement does not inscribe investor-state dispute settlement.

The agreement replicates standard investment clauses known to impact public health policy space and does not follow international best practice regarding investment protection.^44^ It inscribes fair and equitable treatment and full protection and security for investors (Art 13.7) and sets a range of conditions for expropriation including compensation (Art 13.9). Arbitration on the ground of compensation claims in relation to public health, pharmaceuticals and health services is not new.^45,46^ This possibility is not excluded in the agreement, should government regulation (including eg workforce planning and professional regulation) affect investments, or investors' legitimate expectations of NHS or private health contracts. The agreement includes an article on transfers (Art 13.10), which can affect the movement of assets and investments when contracts end, limit profits extracted under NHS contracts or compensation payments if services are returned to public service provision (Art 13.9.1) (Annex 13.A). Art 13.17 allows governments to ensure that investments are taken in a manner sensitive to health, environment, or other regulatory objectives, but does not allow governments to prioritise these objectives over investor interests. While the agreement indicates that the exceptions and exclusions outlined in other chapters take precedence (Art 13.3.1), it does not explicitly protect the right to regulate on health or other matters, as best practice recommends.^44^ Art 13.18 confirms parties’ right to set levels of environmental protection, but no equivalent right exists for other public health goals. Annex 13B excludes non-discriminatory actions for public health, safety, and environment, including with respect to regulation, pricing, supply, and reimbursement for pharmaceuticals, medical devices, and vaccines from indirect expropriation. However, the addition of “except in rare circumstances”, dilutes this commitment to health and constrains the policy space available to governments now obliged to have the burden of proof to show that measures are exceptional and relate to rare circumstances.

In Annex II, the UK has reserved the right to any measures regarding market access and investment liberalisation for public utilities, including health services. For investment, health services receiving any public funding are excluded for market access, national treatment, senior management, board of directors, and performance requirements, but Art 13.7 (Minimum standards), 13.9 (Expropriation and Compensation) (including Annex 13B) and 13.10 (Transfers) still apply. However, considering that the health services arbitration case against Slovakia was based on violation of the transfer article in a bilateral investment agreement,^46^ ignoring this article is not in line with “full protection of the NHS”. This is especially the case as initially Slovakia sought to articulate that health services were outside the scope, but it was decided that as Slovakia had not explicitly excluded health services this was not permissible.^46^ This would suggest that not excluding NHS explicitly makes it vulnerable to challenges on the ground of the transfer article, irrespective of the potential outcome of the challenge. Furthermore, Annex II in the agreement includes investment in privately funded hospitals, restricting scope for government measures affecting investment in private hospitals. This implies the agreement can be used as ground for the protection of investor interests in privately funded hospitals, should the government limit or undermine their role in health services markets. Since no specific clause is set for investment related to NHS services, the agreement could also affect subcontracted services that function under the NHS but do not fall strictly under the definition of ‘UK publicly funded clinical healthcare services’ used in the UK Trade Act.^30^ These services are considered only in relation to their scope for being subject to concessions awarded to the private sector for provision of public infrastructure and services.

### Digital Trade

Chapter 14 (digital trade) excludes audiovisual services, government procurement and financial service suppliers, but not the NHS, despite scope and interest in trade in health-related data^47^ and digital health services. Article (14.2.3) carves out obligations with respect to Cross-border transfer of information (14.10) and Location of computing facilities (14.11) from areas not included under respective chapters on services and investment obligations. However, this leaves uncertainty with respect to NHS data and electronic health records. When compared with the UK, Australia has excluded health data and electronic health records more explicitly. Considering that this is a fast-moving ground with substantial private sector interests, lack of consideration of NHS in this context by the UK government is striking and may undermine public confidence in the NHS.^48^ Future trade negotiations will also have to take into account the rapidly evolving regulatory arena of artificial intelligence (AI), which is understood to provide benefits and risks to public health. In UK-Australia, AI is addressed in the innovation chapter, which we assess below.^49,50^

### Government Procurement

Chapter 16 (government procurement) explicitly references the NHS. The UK makes commitments in Annex 16A. Government procurement is opened above certain monetary thresholds for various public agencies including the NHS, other agencies that are part of the NHS across the UK, and agencies it relies on to fulfil its statutory responsibilities, including local authorities. This implies that NHS has not been excluded “from the scope of the FTA”.

Chapter 16 incorporates a WTO Government Procurement Agreement (GPA) carve-out allowing infringements of procurement rules where this is necessary to protect human, animal and plant life or health, with respect to goods and services of philanthropic institutions and prison labour (Art 16.3). It permits selective and restrictive tendering and clarifies scope for considering environmental, social, and labour issues (16.17). Where specified, Art 16.14.5 allows for the most advantageous tender to be awarded, in contrast to the lowest offer.

Annex 16A lists all institutions and agencies to which Chapter 16 applies under sections A-C with further listing of goods (Section D) and services (Section E) for which commitments are made. There was no requirement to include NHS agencies in section A or Clinical Commissioning Groups (now Integrated Care Boards) in section B, yet the UK has done so. General exclusions for government procurement relate to non-contractual agreements, co-operation between public bodies, and public employment contracts (16.1). Annex 16 A, Section E includes services, where health services have not been included in the first place. However, there is a specific note to indicate that procurement of some human and administrative health services is not included under specific categories (CPC Prov 931, CPC Prov 91122, CPC Prov 870206 and 872). Procurement of goods is covered widely as is procurement of education services.

It is important to note that from the perspective of safeguarding the NHS no commitments were required to be made by the UK for the NHS, yet despite the scope of some commitments being limited, others were included thus restricting the policy space. While the UK could still engage in procurement in alignment with its existing commitments, it would have had wider scope for changing practices if these were not included as part of the agreement.

### Good Regulatory Practice

Chapter 26 (good regulatory practice) is a general obligation and thus also applies to the NHS. The chapter places requirements on governments on development and application of regulatory measures (Acts of Parliament or other statutory instruments).

In Chapter 26, Australia has excluded from the scope regulatory authorities “where those departments are regulating human health”, while the UK has not. However, the UK has excluded public procurement and imposition of taxes and charges from the scope of regulatory measures. The chapter 26.2.3 includes also a typical “interpretational” emphasis that:

“Provisions of this Chapter shall not be construed so as to require a Party to: a) deviate from domestic procedures for identifying its regulatory priorities and preparing and adopting regulatory measures ensuring the levels of protection that the Party considers appropriate to achieve its public policy objectives (including health, safety and environmental goals).”

This needs to be set against the articles (26.5 and 26.6) of the Chapter on Good Regulatory Practices and obligations to require regulatory impact assessment and consultation before implementation of regulatory measures enabling those to be regulated to engage in early stages. There is no such requirement to undertake health impact assessment, for example, to consider the potential adverse impacts of not introducing regulations with obligations following regulatory impact assessment more in line with reduction of regulatory burden than in ensuring regulatory policy space.

### Competition and Consumer Protection and State-Owned Enterprises (SOE)

Chapter 17 commits parties to apply national competition law to private and public commercial activities. The NHS is not exempt. The chapter includes provisions regarding procedural fairness, private rights of action and transparency (17.2-4.). Governments must also maintain laws and regulations for consumer protection, including remedies for services not performed with appropriate care or skill (17.5).

Chapter 18 applies to “activities of state-owned enterprises and designated monopolies of a party that affect trade or investment between the parties.” SOE rules in trade agreements respond to the expansion of state capitalism and ‘competitiveness’” considerations.^51^ However, they also cover domestic trade, non-commercial assistance and services, and are thus relevant to the NHS.

Chapter 18 explicitly excludes audiovisual services and government procurement, but not the NHS. It also makes several exclusions for monetary policy and the regulation of the financial services sector, but not for health or social services. While the NHS may not be the intended focus for SOE provisions, and the exclusions under Art 18.2 (9 and 11) Art 18.9 and Art 18.13 may apply to the NHS and health system, the absence of an NHS exclusion limits legal certainty. This may restrict policy space in the way that services are provided and open the possibility of legal challenges against regulation relevant to the NHS.

### Financial Services

Art 9.1 covers life and non-life insurance. Non-life insurance is anything that protects people, property, or legal liabilities, that is not life insurance. Health insurance is commonly understood to be non-life insurance. The provision does not target the NHS directly, as the NHS is a public healthcare system funded through taxation. Financial services rules in chapter 9 indirectly affect the NHS, as they provide market access to the UK's private healthcare sector.

The UK was among a group of six EU Member States that fully liberalised insurance and related services under the EU's General Agreement on Trade in Services (GATS) schedule under World Trade Organisation (WTO). The UK has replicated this in its independent GATS schedule.^52^ The implications for NHS Foundation Trusts in England that obtain up to 49% of their income from private services, often paid for through health insurance, are not clear.

### Sanitary and Phytosanitary (SPS) Measures and Antimicrobial Resistance (AMR)

The NHS oversees and responds to emerging as well as established public health threats. Chapter 6 is similar to one with provisions in the Comprehensive and Progressive Agreement for the Trans-Pacific Partnership (CP-TPP),^53^ requiring a science-based approach (Art 6.5) and permitting only those trade-restrictive measures, where scientific evidence about harmful impacts exists.^54^ This moves the regulatory approach closer to current US practice rather than the expectation that scientific uncertainty or absence of evidence does not delay measure to limit harm to human, animal and plant health or degrade the environment when a health risk state is identified.^54–55^

Chapter 25 (animal welfare and AMR) does not directly constrain the UK's ability to protect human, animal and plant health, but does little to advance it, particularly where equivalence with the party with the less restrictive measures is promoted (Art 6.7). Chapter 7 (Technical Barriers to Trade) also provides scope for the UK to adopt Australian environmental protection standards where they are less extensive than current UK provisions. Implications of SPS measures (Chapter 26) and several other chapters in the agreement have direct implications for the wider regulatory context of environmental, social and commercial determinants of health beyond the NHS.

### Intellectual Property Rights and Innovation

Chapter 15 includes obligations under national treatment, transparency, and cooperation in IPR and its enforcement. IPR and trade secrets are particularly relevant to new, highly priced medicines and vaccines.^15–17^ This has implications for NHS budgets and the spend on medicines versus other aspects of care. Pharmaceuticals are also important in UK-US negotiations.^56^

Chapter 15 reiterates WTO TRIPS agreement provisions. Art 15.6 mirrors the 2001 Doha Declaration on TRIPS and Public Health including key provisions on access to medicines, and refers to Art 31bis, Appendix and Annex to the TRIPS Agreement. As the Covid-19 pandemic has shown, current agreements do not support rapid access to essential medicines and vaccines by use of compulsory licensing, technology transfer or disclosure of trade secrets^57–58^; contribute to policy coherence with respect to medicines for all; or address unrestrained pricing of medicines and technologies. Art 15.17 mirrors CP-TPP provisions^59^ recognizing the importance of a rich and accessible public domain but provides no mechanism for challenging failure to deposit essential IPR material within it.

The articles on supplementary patent protection and on data exclusivity go beyond WTO law but allow Australia to maintain only 5 years of exclusivity. No time limit is set for supplementary patent protection. The agreement includes explicit research and ‘Bolar’ exceptions expected to support research. This suggests that the IPR section has followed Australian practice. The agreement repeats the TRIPS Agreement on patentable subject matter. Key provisions for pharmaceutical pricing do not extend to the requirements of CP-TPP,^59–60^ however the fact that UK and Australia are now both parties to CP-TPP reduces legal certainty. A separate letter on cooperation concerning medical devices and medicines and their regulation is annexed.^61^

The agreement includes an article on trade secrets. Trade secrets are a preferred pathway for pharmaceutical industries to protect process-related information.^62^ Trade secrets are a concern regarding the safety of clinical trials and for health technology assessments (HTA).^63^ Although Section F, Art 15.48 and 15.49 provide for disclosure for regulatory purposes, assessment of safety of medicines and technology studies, in practice, few mechanisms are available to the NHS, public analysts, or university laboratories to require disclosure of trade secrets. Comprehensive assessment of the cost-effectiveness and safety of new technologies and treatments requires disclosure, so trade secrets may limit public scrutiny, comparison, and wider research on the costs and benefits of pharmaceuticals.^62^ Trade secrets can thus affect both safety and pricing of pharmaceuticals and vaccines.^62–63^

Art 15.69 allows scope for “limited exceptions and limitations” for measures such as freedom of media or revealing misconduct, provided this is done in the public interest. Article 15.3. follows standard measures emphasising the requirement to meet a ‘necessity’ threshold to exercise the right to adopt measures necessary to protect public health and nutrition, rather than this being an entitlement of governments. These measures are further undermined by the requirement to be consistent with the provisions of the chapter. Furthermore, should the government have wanted to ensure that trade secrets would not hinder access to medicines of public health priority or access during emergencies, they could have included this under Art 15.69.

Chapter 20 (innovation) seeks to enhance innovation and best practice especially on new technologies and AI. While transparency regarding trade secrets is important for new medicines, inability to scrutinise AI algorithms raises additional patient safety and equity concerns.^64^ AI is likely to impact the process and evaluation of medical device and technology developments, where independent scrutiny of cost, safety, privacy and added value to the health system remains crucial. While Chapter 20 establishes common frameworks and principles for AI, it provides a placeholder for future negotiations, not binding obligations. Is stated above, AI regulation also falls under digital trade chapters, hence future chapter will need to be assessed together.

## Discussion

We examined the extent to which the UK-Australia FTA safeguards the NHS. Our analysis is grounded in trade negotiation documents and prior research for the identification and framing of potential health implications, including the potential for additional constraints on health policy space. New chapters on innovation (including AI), competition, consumer protection, SOEs, and regulatory cooperation remain more open for interpretation.

While public consultations saw protecting the NHS as a priority,^40^ this is not reflected in the UK government impact assessment.^36^ Despite extensive research on the health impacts of trade agreements,^8–17^ these are not discussed. The impact assessment^36^ discusses health largely in connection with specific environmental impacts. The impact assessment thus fails to provide evidence on potential health implications or how the government has sought to mitigate them. This contrasts with the health impact assessment (HIA) of the CPTPP undertaken by Public Health Wales, which did not examine NHS impacts in detail but highlighted adverse impacts on health, including on health inequalities and people with chronic conditions.^37,65^

The NHS could instead have been removed from the scope of the agreement explicitly and clearly. This was not done. Instead of excluding the NHS completely from the agreement, the negotiated text relies on limited exceptions in particular chapters. The efforts made to safeguard the NHS also failed on comparative grounds as the NHS is not excluded to the same extent as other public policy prerogatives, such as audiovisual services, and in some chapters in comparison to government procurement, financial services, or the environment. The UK did not even mirror Australia's health system exceptions which included protections for local and sub-national not for profit agencies of the type that also provide health and social care and support services in the UK. Increasingly overlapping and complex trade negotiations make guaranteeing the integrity of the specific and limited exclusions applied here vulnerable to negotiations under new chapters and amendments. The NHS can also be affected indirectly through privileges given to private companies as investors, hospital, or other service providers.

Trade agreements impact government's regulatory policy space. They de-regulate, particularly with regard to measures intended to protect workers and the public while introducing new regulatory obligations (eg for protection of IPR and investment).^1^ Regulatory and de-regulatory requirements in trade agreements can produce ‘regulatory chill’ for health-related measures.^65–67^ They also set a cost premium for future governments wishing to re-regulate the NHS or remove private sector competition from NHS activities.

Our findings complement studies on negotiation practices,^68^ trade agreements and pharmaceutical policy^69^ as well as studies emphasising the emerging role of more bilateral and WTO + negotiations.^70^ The also give support to prior studies of how trade agreements enhance multinational corporate power, interests and regulatory chill above health and other public interests.^71–73^ Our analysis indicates that Brexit did not bring improved consideration of the NHS in trade agreements in practice and that accountability for claims of governments will need to be followed through trade negotiations. Finally, it focuses on the NHS but is mindful of the consequences on social, environmental, and commercial determinants of health resulting from provisions in several chapters.

The published UK government impact assessment provides false reassurance that the NHS is out of scope of the UK-Australia agreement when this is not the case. It is also important to understand how the then Secretary of State's assurance in the House of Commons that the NHS is excluded from the agreement came about, given that our analysis does not confirm this. While the Health and Social Care Act 2022 and revised procurement guidance may limit the extent of NHS vulnerability, if the UK government renegotiates the FTA, it will need to step up measures that go beyond rhetoric to safeguard the NHS and its infrastructure as a universal, comprehensive, tax-funded health system, not just a popular brand that provides a range of publicly-funded clinical healthcare services.

## Conclusions

Our analysis indicates that, in contrast to claims before negotiations and in the summary of the impact assessment, the NHS is not out of the scope of the negotiated FTA. It highlights the need to focus on what has been negotiated in practice. Furthermore, greater care has been taken to protect other public policy prerogatives such as audiovisual services and public broadcasting, than universal, comprehensive healthcare free at the point of use. There is no explicit general exclusion for the NHS from the scope of the agreement. Commitments taken on service trade, investment, digital trade, procurement, good regulatory practice, SOEs, financial services, SPS and antimicrobial resistance, IPRs and innovation stand to affect the NHS directly or indirectly. The risks increase if the UK adopts the same approach to trade and health in future trade negotiations or in partial agreements and side-deals that are increasingly popular in the current era of heightened trade conflict.
